# Repetitive mRNA vaccination is required to improve the quality of broad-spectrum anti–SARS-CoV-2 antibodies in the absence of CXCL13

**DOI:** 10.1126/sciadv.adg2122

**Published:** 2023-08-04

**Authors:** Marne Azarias Da Silva, Pierre Nioche, Calaiselvy Soudaramourty, Anne Bull-Maurer, Mounira Tiouajni, Dechuan Kong, Ouafa Zghidi-Abouzid, Morgane Picard, Ana Mendes-Frias, André Santa-Cruz, Alexandre Carvalho, Carlos Capela, Jorge Pedrosa, António Gil Castro, Paul Loubet, Albert Sotto, Laurent Muller, Jean-Yves Lefrant, Claire Roger, Pierre-Géraud Claret, Sandra Duvnjak, Tu-Anh Tran, Kenzo Tokunaga, Ricardo Silvestre, Pierre Corbeau, Fabrizio Mammano, Jérôme Estaquier

**Affiliations:** ^1^ INSERM-U1124, Université Paris Cité, Paris, France.; ^2^ Structural and Molecular Analysis Platform, BioMedTech Facilities INSERM US36-CNRS UMS2009, Université Paris Cité, Paris, France.; ^3^ Université de Tours, INSERM, UMR1259 MAVIVH, Tours, France.; ^4^ Joint Research Center for Human Retrovirus Infection, Kumamoto University, Kumamoto, Japan.; ^5^ Department of Pathology, National Institute of Infectious Diseases, Tokyo, Japan.; ^6^ CHU de Québec-Université Laval Research Center, Québec City, Québec, Canada.; ^7^ Life and Health Sciences Research Institute (ICVS), School of Health Sciences, University of Minho, Braga, Portugal.; ^8^ ICVS/3B’s-PT Government Associate Laboratory, Braga/Guimarães, Portugal.; ^9^ Department of Internal Medicine, Hospital of Braga, Braga, Portugal.; ^10^ Service des Maladies Infectieuses et Tropicales, CHU de Nîmes, Nîmes, France.; ^11^ Service de Réanimation Chirugicale, CHU de Nîmes, Nîmes, France.; ^12^ Urgences Médico-Chirugicales Hospitalisation, CHU de Nîmes, Nîmes, France.; ^13^ Service de Gérontologie et Prévention du Vieillissement, CHU de Nîmes, Nîmes, France.; ^14^ Service de Pédiatrie, CHU de Nîmes, Nîmes, France.; ^15^ Institut de Génétique Humaine, UMR9002 CNRS-Université de Montpellier, Montpellier, France.; ^16^ Laboratoire d’Immunologie, CHU de Nîmes, Nîmes, France.

## Abstract

Since the initial spread of severe acute respiratory syndrome coronavirus 2 infection, several viral variants have emerged and represent a major challenge for immune control, particularly in the context of vaccination. We evaluated the quantity, quality, and persistence of immunoglobulin G (IgG) and IgA in individuals who received two or three doses of messenger RNA (mRNA) vaccines, compared with previously infected vaccinated individuals. We show that three doses of mRNA vaccine were required to match the humoral responses of preinfected vaccinees. Given the importance of antibody-dependent cell-mediated immunity against viral infections, we also measured the capacity of IgG to recognize spike variants expressed on the cell surface and found that cross-reactivity was also strongly improved by repeated vaccination. Last, we report low levels of CXCL13, a surrogate marker of germinal center activation and formation, in vaccinees both after two and three doses compared with preinfected individuals, providing a potential explanation for the short duration and low quality of Ig induced.

## Teaser

Three doses of mRNA vaccine are required to develop a strong and long-lasting humoral response against SARS-CoV-2 variants.

## INTRODUCTION

Since the initial SARS-CoV-2 (severe acute respiratory syndrome coronavirus 2) pandemic related to the Wuhan strain (*1*), several viral variants have emerged. These variants, particularly Beta (B.1.351), Delta (B.1.617.2), and, more recently, diverse Omicron subtypes, represent a major challenge for immune control, especially in the context of vaccination. Most of the mutations that differentiate these strains from the original isolate are localized in the two domains of the spike (S) protein shown to be targeted by neutralizing antibodies (*2*–*4*): the receptor binding domain (RBD) that interacts with the angiotensin II (ACE2) receptor and the N-terminal domain (NTD).

Current vaccines, such as those manufactured by Pfizer/BioNTech (BNT162b2) and by Moderna/National Institute of Allergy and Infectious Diseases (mRNA-1273), encode for an S protein whose sequence is similar to the early Wuhan-Hu viral isolate. The emergence of viral variants has consequently challenged vaccine effectiveness. Initial reports have shown lower levels of recognition of Beta and Delta variants, even after the second dose of vaccine (*5*–*8*). The recently emerged Omicron variants were reported to be less efficiently neutralized than the Wuhan-Hu strain by immunoglobulin G (IgG) from vaccinated individuals even after a third dose (*9*–*15*) and by therapeutic neutralizing antibodies (*16*–*18*).

Beyond neutralizing antibody, it has been shown that Fc effector mechanisms including antibody-dependent complement deposition, antibody-dependent neutrophil phagocytosis, and antibody-dependent cellular cytotoxicity responses may contribute in the control of viral dissemination by clearing viral-infected cells and limiting disease severity (*19*, *20*). We recently demonstrated that the amount of IgG capable to recognize the Wuhan-Hu S-protein on cell surface of transfected cells are lower in patients with severe coronavirus 2019 (COVID-19), and this was associated with frequent CD4 T cell apoptosis (*21*, *22*). Furthermore, it has been suggested that antibody cellular effector functions induced by mRNA vaccine are preserved despite the loss of Omicron neutralization, indicating a disconnection between the requirements for quantity and quality of antibodies for the two functions (*23*). An incomplete natural immunity against variants has been also reported in convalescent individuals (*24*), who displayed lower quality of Fc-mediated antibody responses compared to individuals vaccinated with two doses of mRNA-1273 (*25*). Nevertheless, convalescents individuals boosted with vaccine are better protected against reinfection than vaccinated alone with two doses of vaccine (*26*–*28*).

While most of the studies have assessed the role of neutralizing IgG, little is known about mucosal humoral response induced by vaccines. Studies including ours have shown that, early after SARS-CoV-2 infection, a dominant IgA humoral response is induced against the nucleocapsid (N) and S proteins (*29*, *30*). The presence of IgA in vaccinated individuals would be extremely important in the event of further contact with the virus, particularly during the first days of infection. The IgA in the mucosal tissue could limit viral dissemination and disease outcome, but little is known on the delay and durability of this response in individuals, with or without previous infection, who have received a vaccine boost.

Although the beneficial effect of vaccination is well established (*31*, *32*) and vaccinees mount a competent humoral response against SARS-CoV-2 (*33*–*37*), repetitive vaccination campaigns have been necessary to maintain an efficient humoral response capable of preventing severe forms and hospitalization. The requirement for repetitive doses to improve humoral response and cross-reactivity suggests a short half-life of the antibodies induced in the absence of boost and probably a low avidity response. Paradoxically, few studies have determined the avidity of Ig in vaccinated individuals, which reflect antibody maturation following germinal center (GC) formation (*38*, *39*). In this context, measuring the level of chemokine (C-X-C motif) ligand 13 (CXCL13) in the blood may represent an interesting biomarker of GC activation in humans associated with protective humoral response following vaccination (*40*–*42*).

In this study, we evaluated the humoral response of vaccinated individuals, some of whom had also been infected during the first wave of SARS-CoV-2 in 2020, before vaccines became available. By analyzing both the quantity and quality of IgG and IgA, our results demonstrated that the amount and persistence of Ig were higher in individuals previously exposed to the virus and boosted with vaccine compared to vaccinated-only individuals. Three doses of mRNA vaccine were required to improve the quantity, quality, and cross-reactivity against Beta and Omicron variants. We found difference in recognition among Omicron subtypes, between vaccinees only and preinfected individuals. Thus, mRNA vaccine induced Ig capable to recognize variant S proteins expressed on cell surface that is of major importance for Fc-mediated function by vaccines. While CXCL13 levels are high during the acute phase of SARS-CoV-2 infection, vaccine administration, even after the third dose, has no impact on the levels of CXCL13 detected in the plasma. This result may help to explain why booster vaccination induces a potent humoral response in previously infected patients compared with vaccinated-only individuals who require at least three doses of vaccine to reach similar levels of humoral response. Thus, our work provides a framework to explain the need for repeated immunizations to provide stronger and longer-lasting humoral responses, which might contribute to controlling viral dissemination even against variants of concern.

## RESULTS

### Three mRNA vaccinations are required for IgG and IgA responses similar to those of convalescent and boosted individuals

**Fig. 1.**
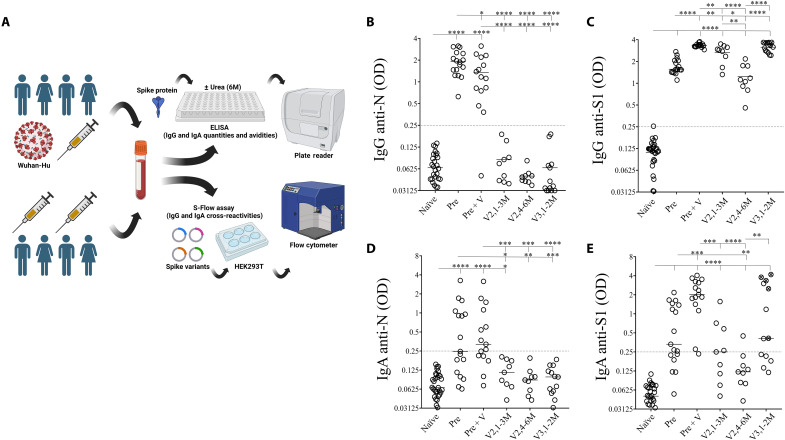
IgG response against the N and spike proteins in convalescents and vaccinated individuals. (**A**) Plasma from healthy donors (naïve) convalescent individuals (Pre), convalescent individuals boosted with vaccine (Pre + V), vaccinees after two doses either at months 1 to 3 (V2,1-3M) or months 4 to 6 (V2,4-6M), and after three doses at months 1 to 2 (V3,1-2M) were diluted to 1/400. (**B** to **E**) Plasma from healthy donors (naïve) convalescent individuals (Pre), convalescent individuals boosted with vaccine (Pre + V), vaccinees after two doses either at months 1 to 3 (V2,1-3M) or months 4 to 6 (V2,4-6M), and after three doses at months 1 to 2 (V3,1-2M) were diluted to 1/400. (B) and (C) Specific immunoglobulin G (IgG) and (D) and (E) IgA were tested against the nucleocapsid (N) and spike (S1) proteins. Optical density (OD) is shown. Each circle represents one individual. Lines represent median values. Dashed lines represent antibody specificity (OD = 0.25) in comparison with IgG and IgA from healthy donors. Statistical analysis was performed using a Mann-Whitney *U* test (*P < 0.05; **P < 0.01; ***P < 0.001; ****P < 0.0001). (C) and (E) Symbols with a cross represent individuals who received at least one dose of mRNA-1273, whereas open symbols represent individuals who only received BNT162b2 in the vaccination scheme.

To determine the impact of mRNA vaccination boosts, we analyzed humoral responses in different groups of donors (Fig. 1A). Individuals included (i) nonvaccinated convalescent individuals, from whom samples were collected 6 months after SARS-CoV-2 infection (Pre; *n* = 17); (ii) convalescent individuals vaccinated with BNT162b2 (1 to 3 months after vaccination, Pre + V; *n* = 15), so called hybrid immune responders; and (iii) individuals only vaccinated with two doses (*n* = 9, samples collected at two time points: V2,1-3M, 1 to 3 months after vaccination and V2,4-6M, 4 to 6 months after vaccination) or (iv) with three doses of mRNA vaccines (1 to 2 months after vaccination, V3,1-2M; *n* = 13) and a group of naïve individuals as a negative control (naïve, *n* = 31). All convalescent individuals were infected between March and December 2020, when only the original strain and the Alpha variant were circulating in Europe. As indicated in Table 1, five individuals received the Moderna vaccine for their third dose, whereas all the others only received the Pfizer formulation. We first assessed the levels of specific antibodies against the S1 and N antigens by ELISA (enzyme-linked immunosorbent assay) as previously described (*16*). This latter antigen was used as a marker to follow individuals that may have been infected with SARS-CoV-2. The optical density (OD) values of the ELISA performed with patients’ plasma are shown in Fig. 1 (B to E). As expected, anti-N IgG were detected in convalescent individuals irrespective of their vaccination status but not in vaccinated-only individuals (Fig. 1B) nor in the naïve group. Although the OD values of anti-N IgG antibodies were significantly different (*P* = 0.048), the levels of anti-S1 IgG were clearly higher in convalescents boosted with a vaccine dose (hybrid immunity, Pre + V: 3.37 ± 0.22) compared with nonvaccinated convalescent individuals (Pre: 1.57 ± 0.43, *P* < 0.0001) both at 1/400 (Fig. 1C) and 1/800 dilutions (fig. S1). This difference may in part be due to the longer time after exposure of the convalescent individuals (6 months) as compared to convalescent and vaccinated individuals (1 to 3 months). Only one individual had anti-N IgG antibodies below the positive threshold. Notably, the levels of specific anti-S1 IgG, 1 to 3 months after the second dose, remained lower (V2,1-3M: 2.88 ± 0.73) than those observed in convalescents receiving one dose of vaccine (Pre + V: 3.37 ± 0.22, *P* = 0.0035; Fig. 1C). The OD values of anti-S1 IgG were lower 4 to 6 months after the second dose (V2,4-6M: 1.12 ± 0.54) and increased again, boosted by the third dose (V3,1-2M: 3.24 ± 0.48).

**Table 1.** Characteristics of individuals included in this study. M, male; F, female.

Groups/vaccine	*N*	Pfizer (BNT162b2)	Moderna (mRNA-1273)	Age, years Median	Gender	
				[Range]	M	F
Pre: Convalescents 6 months after infection	17			67 [52–87]	11	6
Pre + V: Convalescents + vaccine (1 to 3 months after vaccination)	15	15		56 [25–81]	5	10
V2,1-3M: Vaccinated two doses (1 to 3 months after vaccination)	9	9		46 [12–85]	5	4
V2,4-6M: Vaccinated two doses (4 to 6 months after the vaccination)	9	9		57 [28–85]	4	5
V3,1-2M: Vaccinated three doses (1 to 2 months after the vaccination)	13	8	5	54 [12–85]	7	6

Having observed differences in the IgG response between vaccinated individuals and those previously infected with SARS-CoV-2, we then compared their IgA responses. We and others have shown that humoral response against the S protein also includes IgA (*21*, *43*–*45*). Furthermore, IgA were reported to dominate the early antibody response to SARS-CoV-2 (*21*, *29*). The presence of IgA, boosted by the mRNA vaccine, could be of importance, since IgA is the most abundant antibody isotype in the mucosa, where these antibodies provide the first line of immune defense against pulmonary viral infections (*46*). Like IgG, specific IgA were assessed by ELISA at the same dilution in plasma (1/400). In convalescent individuals (Pre), we found specific IgA against the N (8 of 17) and S1 (10 of 17) proteins (Fig. 1, D and E, respectively). The OD values of IgA (Fig. 1, D and E) were significantly lower than those observed for the IgG (Fig. 1, B and C). This was observed both for Ig anti-N (Pre, *P* < 0.0001 and Pre + V, *P* = 0.0209) and for anti-S (Pre, *P* = 0.0002 and Pre + V, *P* = 0.0027). IgA response against S was improved by mRNA boost (hybrid patients, Pre + V: 1.93 ± 1.20) compared to convalescent individuals without vaccination (Pre, 0.33 ± 0.69; Fig. 1E). Our results highlighted that two doses of mRNA induce low levels of IgA against S1 (V2,1-3M: 4 of 10 individuals were responders), which declined after 4 to 6 months (V2,4-6M: 1 of 10 individuals were responders). After the third dose, more than half of vaccinated individuals (8 of 13) developed high level IgA responses (Fig. 1E). Of interest, IgA levels were higher in individuals who received one dose of mRNA-1273 compared to individuals who received only BNT162b2 (OD, 3.35 ± 1.5 and 0.22 ± 1.1, respectively, *P* = 0.01), whereas this difference was lower for IgG response (OD: mRNA-1273, 3.63 ± 0.14 and BNT162b2, 2.8 ± 0.41, *P* = 0.01). Together, our results confirm the need for repeated administration of mRNA vaccines, with at least three doses to induce a humoral response against S like that seen in individuals previously infected by SARS-CoV-2 and boosted with mRNA vaccine.

### Repeated mRNA vaccinations improve IgG and IgA responses to recognize viral variants although Beta and Omicron BA.1 remain of concern

In addition to neutralization, antibodies contribute to clearing viral-infected cells through different mechanisms limiting viral dissemination and have recently been described as participating in immune defense against SARS-CoV-2 (*19*). To assess the recognition of viral proteins by antibodies present in patients’ plasma, the S-Flow assay relies on transfected cells expressing the S protein on the cell surface using flow cytometry (*16*, *47*). Transfection of plasmids encoding the S protein does not require biosafety level 3 confinement and allows to test recent isolates without the need for replication-competent virus isolation. Before using transfected cells, we assessed whether antibodies present in the plasma of vaccinees and convalescent individuals were capable to recognize viral antigens on the surface of Wuhan-Hu infected cells (fig. S2). Whereas we clearly detected infected cells compared to uninfected cells, one cannot formally assume that S was the sole antigen present on cell surface.

**Fig. 2.**
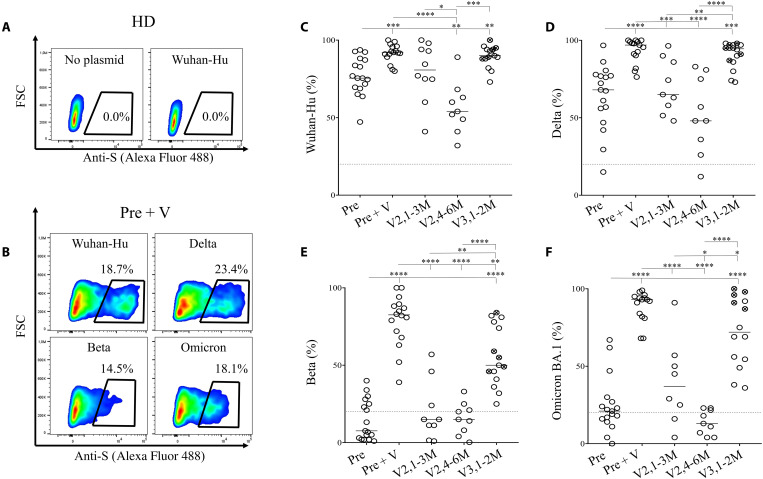
IgG cross-reactivity against viral variants in convalescents and vaccinees. (**A** and **B**) Representative S-Flow assay. HEK293T cells either nontransfected or transfected with a plasmid encoding for the S protein were either labeled with (A) plasma from a healthy donor (HD) or (B) with plasma from a convalescent individual boosted with a vaccine dose (Pre + V). The percentages of cells recognized by specific IgG were detected by flow cytometry are shown for each variant. (**C** to **F**) Plasma from individuals, assessed in Fig. 1, were monitored for their capacity to recognize the different S variants including (C) Wuhan-Hu, (D) Delta, (E) Beta, and (F) Omicron. Relative percentages were calculated as follows: (% of IgG from plasma individuals − % of secondary IgG alone/% of anti-S2 mAbs − % of secondary IgG alone)*100. Each circle represents one individual. Lines represent median values. Statistical analysis was performed using a Mann-Whitney *U* test (*P < 0.05; **P < 0.01; ***P < 0.001; ****P < 0.0001). Symbols with a cross represent individuals who received at least one dose of mRNA-1273, whereas open symbols represent individuals who only received BNT162b2 in the vaccination scheme. Dot plots show forward size scatter (FSC) against anti-S detection.

We then analyzed the ability of IgG to cross-recognize viral variants by expressing S proteins on the cell surface upon transfection. To normalize the data for each variant, the results were expressed as the percentages of cells recognized by the patient’s plasma, while a specific monoclonal antibody recognizing transfected cells against S2 was used as a positive control and attributed a value of 100% (fig. S3). Figure 2 shows the specific detection of S proteins by flow cytometry. Plasma from a healthy donor (Fig. 2A) did not recognize transfected cells, whereas plasma from a vaccinated convalescent individual recognized the S proteins of four viral variants, expressed on the cell surface (Fig. 2B). As expected, plasma from all convalescent individuals recognized the Wuhan-Hu strain (Pre: 75.5 ± 12.3%; Fig. 2C), whereas the percentages of S-Flow decreased for Delta (Pre: 68 ± 21%; Fig. 2D) and were extremely low for Beta and Omicron BA.1 (Pre: 7.6 ± 13.1 and 21 ± 18.2%, respectively; Fig. 2, E and F). Thus, convalescent individuals displayed low cross-reactivity. In contrast, convalescent individuals boosted with the mRNA vaccine (Pre + V) developed IgG that recognized all variants including Beta and Omicron. Vaccinated-only individuals demonstrated specific IgGs against the Wuhan-Hu (84.5 ± 19.5%) and Delta (65 ± 17.3%) after the second dose (V2,1-3M; Fig. 2, C and D). The percentages of S-Flow were lower for Beta (15 ± 20.1%) and BA.1 (37 ± 27.3%) compared to convalescent vaccinated individuals (Fig. 2, E and F). However, IgG reactivity markedly decreased at months 4 to 6 (V2,4-6M), including for the Wuhan-Hu strain, and was particularly low for Beta and BA.1 (17.5 ± 9.3 and 13 ± 7.9%, respectively; Fig. 2, E and F). Boosting humoral response with a third dose not only increased the levels of specific IgG against Wuhan-Hu and Delta (Fig. 2, C and D) but also induced significantly higher humoral responses against Beta and BA.1 (Fig. 2, E and F). Patients having received a dose of mRNA-1273 vaccine were better responders against BA.1 than those who received only three doses of BTN162b2 vaccine (S-Flow, 96 ± 5.3 versus 52.7 ± 18.6%, respectively, *P* = 0.004).

**Fig. 3.**
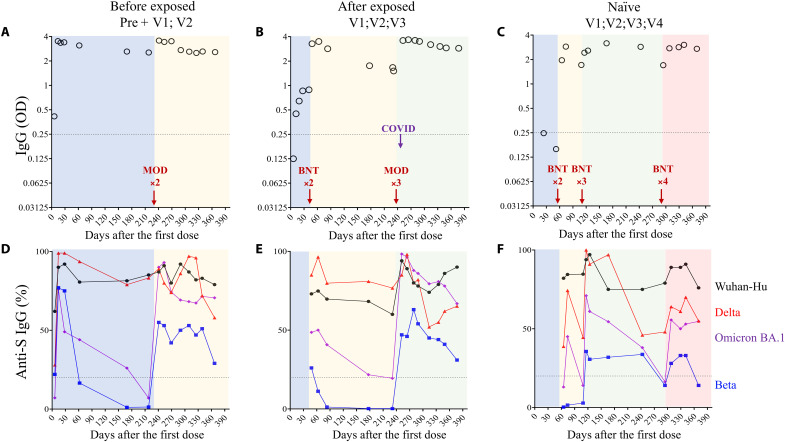
Longitudinal analysis of IgG response either in convalescents boosted with mRNA vaccine or vaccinees. (**A** to **C**) ELISA was used to assess specific IgG response against S protein. Plasma were diluted to 1/400. Circles represent blood samplings at different time points after vaccination (first dose). Red arrows represent dates of vaccine boosts [BioNTech (BNT): BNT162b2 or Moderna (MOD): mRNA-1273]. In (B), the date of severe acute respiratory syndrome coronavirus 2 (SARS-CoV-2) infection is indicated. In (C), uninfected SARS-CoV-2 individual (naive) is shown. OD is shown. Dashed lines represent antibody specificity (0.25). COVID, coronavirus disease. (**D** to **F**) S-Flow assay was used to detect specific IgG cross-reactivity against viral variants. Thus, plasma from the same individuals at the same time points were tested against transfected cells expressing either the Wuhan-Hu spike strain (black circles), Delta variant (red triangles), Beta variant (blue squares), or Omicron variant (violet diamonds). Results are expressed as the percentages of specific IgG recognizing transfected cells by flow cytometry.

We had the opportunity to obtain sequential samples over more than 1 year after vaccination from three individuals (Fig. 3) including one convalescent individual who had been vaccinated (panel A), one vaccinated individual who was infected after the third dose (panel B), and a third one who had received four doses of the mRNA vaccine (panel C). For the three individuals, we found high levels of IgG after two exposures (natural or vaccine). After the third exposure, the levels of specific IgG antibodies plateaued for at least 6 months and were boosted in the third individual after an additional dose.

Assessing variant recognition, we found that, in the individual preinfected with SARS-CoV-2 and boosted with one dose of vaccine, IgGs were capable of recognizing all four viral strains (Fig. 3D). However, the levels of antibodies capable of recognizing Beta and BA.1 markedly decreased in comparison to Wuhan-Hu and Delta until the booster (Fig. 3D). The second dose improved humoral response against the four strains, although IgGs recognizing Beta remained lower. Likewise, in the second individual (Fig. 3E), IgG induced by vaccination recognized Wuhan-Hu and Delta strains, whereas the cross-reactivity of IgG against Beta and BA.1. rapidly declined. The third dose improved cross-reactivity against all four strains, although recognition of Beta was lower compared to the other strains. In this individual, who was infected after vaccination, recognition of the BA.1 variant rose markedly and reached the same level as for the Delta variant, which had been predominant before (Fig. 3E). Last, in the third individual (Fig. 3F), two doses of vaccine were not enough to generate IgG capable of recognizing the Beta strain (Fig. 3F). After the third dose, an increase was observed but Beta and BA.1 recognition declined over the 6-month interval (Fig. 3F). Despite an additional dose (Fig. 3F), IgG did not reach higher levels against Beta, and the percentages of cross-reactivity against Beta, Delta, and BA.1 remained lower compared to Wuhan-Hu and did not exceed 50% (Fig. 3F).

**Fig. 4.**
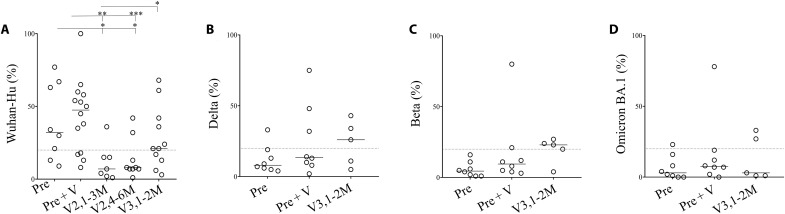
IgA cross-reactivity against viral variants in convalescents and vaccinees. (**A**) Wuhan-Hu, (**B**) Delta, (**C**) Beta and (**D**) Omicron. Percentages of specific IgA detecting variants by flow cytometry (S-Flow assay) are shown. Plasma from V2 were not tested against Delta, Beta, and Omicron due the low levels of IgA detected by ELISA. In (B) to (D), only IgA responders against Wuhan-Hu were tested. Relative percentages were calculated as follows: (% of IgA from plasma individuals − % of secondary IgA alone/% of anti-S2 mAbs − % of secondary IgA alone)*100. Each circle represents one individual. Lines represent median values. Statistical analysis was performed using a Mann-Whitney *U* test (*P < 0.05; **P < 0.01; ***P < 0.001).

We then assessed IgA cross-reactivity (Fig. 4). In some convalescent individuals and in most of those who received a boost (10 of 14), IgA recognized the Wuhan-Hu (Fig. 4A). However, while individuals with two doses of mRNA vaccine had low levels of IgA, half of the vaccinees who had received a third dose (V3,1-2M) developed specific IgA (Fig. 4A). We then assessed variant cross-reactivities in this subgroup of IgA responders. Overall, we observed a low cross-reactivity with some individuals, either convalescents boosted with the vaccine (Pre + V) or vaccinees who had received three doses (V3,1-2M), maintained a cross-reactivity against Delta (Fig. 4B), but very few recognized Beta and BA.1 (Fig. 4, C and D). IgA from convalescents (Pre) were unable to recognize Delta, Beta, or BA.1.

Thus, these results demonstrated the efficacy that can be reached by repeated administrations of mRNA vaccine to induce IgG and IgA that may contribute to the elimination of infected cells. However, without natural infection, specific IgG do not persist for long time, low levels of IgA are produced, and one of the main concerns is the low recognition of Beta and BA.1 variants.

### Structural analysis of RBD and NTD reveals potential regions in variants that may impact antibody recognition

**Fig. 5.**
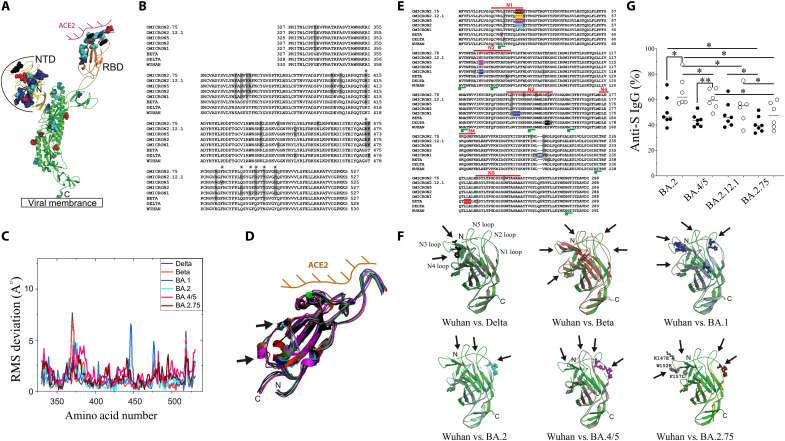
Structural comparison of SARS-CoV-2 variants and Omicron BA.2 subtype IgG cross reactivities. (**A**) One monomer of S protein where the angiotensin II (ACE2) interaction with the receptor binding domain (RBD) is indicated in pink and N-terminal domain (NTD) area exposed to the solvent is indicated by a black line. (**B**) Sequence alignment of the RBD domains where mutations in viral variants are indicated in gray in comparison to the Wuhan sequence. (**C**) Root mean square (RMS) deviation from the RBD structural alignment against the Wuhan-Hu structure [Protein Data Bank identifier (PDB ID): 7L2E]. All RBD structures are in the down state (PDB ID used: 7Q9I, Beta; 7SO9, Delta; 7TM0, Omicron BA.1; 7UB0, Omicron BA.2; 7XNS Omicron BA.4/5; and 7YR1, Omicron BA.2.75). The structure from Omicron BA.2.12.2 was not yet validated. (**D**) Structural superimposition of the Wuhan RBD with the different variants. The main differences are indicated by arrows. (**E**) Sequence alignment of the NTD where insertion/deletion in the Omicron variants are in color. The other mutations are indicated in gray. (**F**) Superimposition of the Wuhan-Hu NTD with Delta, Beta, Omicron BA.1, Omicron BA.2, Omicron BA.4/5, or Omicron BA.2.75 NTDs. Arrows indicate large structural variations of solvent exposed loops. (**G**) IgG cross-reactivity against Omicron BA.2 subtypes. Plasma from either preexposed vaccinated individuals (Pre + V; open circles) or individuals vaccinated either with three doses of vaccine (V3,1-2M; black circles) were monitored for their capacity to recognize the Omicron BA.2 variants. The relative percentages were calculated as described in Fig. 2. Each circle represents one individual. Statistical analysis was performed using a Mann-Whitney *U* test (*P < 0.05; **P < 0.01).

Several studies have previously described the impact of mutations on viral infectivity and escape from recognition by monoclonal antibodies (mAbs) used in therapy, suggesting the importance of the RBD as well as the NTD (Fig. 5A) (*2*–*4*). Delta RBD mutations, which do not include N501Y, are L452R and T478K, and Omicron (BA.1) has seven mutations that map to the ACE2 binding footprint (K417N, S477N, Q493R, G496S, Q498R, N501Y, and Y505H; Fig. 5B, amino acids are indicated by an “*”). These mutations are mainly conserved in the other BA.2 variants of concern. Beta has only three mutations in the RBD compared to Wuhan-Hu (K417N, E484K, and N501Y; Fig. 5B), which are also present in the Omicron subtypes. These minimal differences in the Beta RBD have been previously reported to substantially decrease neutralization by class I and class II monoclonal antibodies (*16*, *48*, *49*). By superimposing the variant structures onto the Wuhan-Hu RBD structure in the down state (not interacting with ACE2), the structural differences are centered around amino acids 365 and 380 (Fig. 5, C and D). These variations almost disappear entirely when the RBD is in the “up” conformation and interacting with the ACE2 receptor (fig. S4, A and B). The lower capacity of IgG to recognize Beta in comparison to Wuhan-Hu spike proteins in transfected cells is unlikely to be due to the differences in the RBD alone. The NTD region is the second most variable domain in which a supersite was reported flanked by glycans that also contribute in neutralizing SARS-CoV-2 infection (*6*, *50*–*53*). Whereas no insertion or deletion of amino acids have been observed within RBD in any variants so far, they have been observed in the NTD (Fig. 5E). These deletions affect the main solvent-exposed loops in the Beta (due to a deletion localized inside the structure affecting the five external loops) and in the Omicron variants (Fig. 5F) compared to the Wuhan-Hu and Delta strains (Fig. 5F). All the BA.2 variants compared to BA.1 contained a deletion in the N1 loop as well the absence of a glycan (NxT/S sequence is replaced by NxI) (Fig. 5E). The variation in the Delta structure was only observed around the single insertion at amino acids E156 and F157. This was further confirmed by plotting the root mean square (RMS) deviation of those six superimpositions compared to the insertion/deletion positions (fig. S4, C to F). On the other hand, insertion and/or deletion in the Beta and Omicron variants that induced large structural variations on external loops (Fig. 5F) could alter IgG recognition of the S protein expressed on cell surface.

We assessed in the S-Flow assay (*21*) the impact of such conformational changes using a mAb that recognizes the NTD domain. This mAb recognized cells expressing the Wuhan-Hu strain (fig. S5A). On the other hand, cells expressing Delta were not recognized by this clone (4A8) due to the deletion in the mAb binding site. Consistent with the notion that mutations/deletions in the NTD affect cross-reactivity, neither Beta- nor BA.1-expressing cells were recognized by this mAb (fig. S5). Therefore, considering that the main differences of BA.2 subtypes compared to BA.1 are related to the N1, N2, and N3 loops of the NTD (Fig. 5F), we hypothesized that such differences may provide a support for immune escape. Analyses of humoral response against BA.2 sublineages (BA.4/5, BA.2.12.1, and BA.2.75) revealed that IgG from vaccinees (V3,1-2M) were less capable to recognize BA.2 than BA.1 variant expressed on the cell surface of transfected cells (BA.2, 50.8 ± 11% and BA.1, 71.6 ± 23%, *P* = 0.03, respectively; Figs. 2F and 5G). We also observed that IgG from convalescents boosted with the vaccine (Pre + V) recognized less efficiently BA.2 than BA.1 (66.8 ± 12 and 88.5 ± 10%, *P* = 0.007, respectively; Figs. 2F and 5G). Recent works suggest that Omicron BA.2 variants are more resistant to neutralization than BA.2 (*54*, *55*). Comparing BA.2 subtypes, BA.4/5 are less recognized than BA.2 by IgG from vaccinees than individuals preinfected and vaccinated (BA.2: V3,1-2M, 50.8 ± 11% versus Pre + V, 66.8 ± 12%, *P* = 0.02 and BA.4/5: V3,1-2M, 43.7 ± 5% versus Pre + V, 60 ± 8%, *P* = 0.001, respectively; Fig. 5G). However, BA.2.12.1 and BA.2.75 sublineages are the main variants of concern even for preinfected vaccinees (Pre + V). Thus, these percentages decreased to 53.9 ± 13 and 47.8 ± 10% in this group and were not significantly different from those observed in vaccinees (V3,1-2M, 48.2 ± 10 and 40.7 ± 6%; Fig. 5G).

Our results support the idea that mRNA vaccines induce IgG whose recognition is also affected by the structural modifications in the NTD region, which, in addition to the RBD, is the most variable regions in SARS-CoV-2. Note that BA.2.75, which displays three additional mutations in the third loop of the supersite (*51*), is the least recognized BA.2 sublineage. This may be indicative of immune pressure and represent one possible mechanism of immune escape. The lower recognition of the S proteins expressed on cell surface would limit cellular effector functions mediated by antibodies and reduce the control of SARS-CoV-2 variants, particularly in the context of BA.2.12.2 and BA.2.75 variants that recently emerged worldwide.

### Three mRNA vaccinations allow to produce antibodies with similar affinities as those from convalescent and boosted individuals

**Fig. 6.**
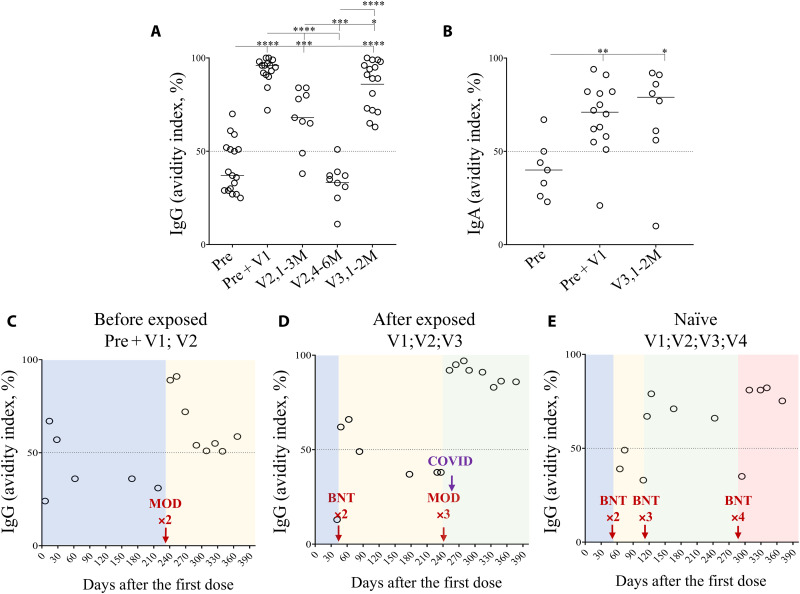
Avidity of IgG and IgA against the spike protein in convalescents and vaccinated individuals. The avidity index (AI; OD with 6M urea/OD without urea × 100) was assessed by ELISA from plasma of individuals as described in Fig. 1. Plasma are diluted to 1/400. Thus, the AIs of (**A**) specific IgG and (**B**) IgA are shown. Dashed lines represent high avidity levels (indexes with a value above 50% are considered to be high, those between 31 and 49% was considered as intermediate, and values below 30% are considered to be low). Lines represent median values. (**C** to **E**). AIs of IgG from plasma of individuals described in Fig. 3 were measured longitudinally. Statistical analysis was performed using a Mann-Whitney *U* test (*P < 0.05; **P < 0.01; ***P < 0.001; ****P < 0.0001).

The progressive loss in the levels and cross-reactivities of specific IgG and IgA requiring repeat vaccinations to maintain their efficacy prompted us to explore the avidity of Ig induced by the mRNA vaccine. The avidity of antibodies reflects the quality and strength of the antibody-antigen complex resulting from the Ig maturation process (*38*, *56*). However, little attention has been paid to the avidity of anti-S antibodies during COVID-19 vaccination. Figure 6A shows the avidity index (AI) of IgG against the Wuhan-Hu S1 protein using a denaturing urea treatment (6M). The AIs of antibodies with values more than 50% are considered as high (*56*). Our results highlighted that a vaccine boost markedly improves the AI of IgG (Pre + V, 95.4 ± 7.1%) compared to IgG from convalescent individuals (Pre, 37 ± 13.9%; Fig. 6A). In vaccinees, the AI of IgG from individuals receiving three doses of vaccine (V3,1-2M: 90 ± 13%) was very high, reaching the same level of convalescent and boosted individuals. This index was higher compared to IgG from individuals receiving only two doses of vaccine either early after vaccination (V2,1-3M: 68 ± 16%) or later (V2,4-6M: 35 ± 10.8%; Fig. 6A).

By plotting the AIs against the percentages of S-Flow recognition, we found a strong association in individuals previously infected and boosted with vaccine (Pre + V; fig. S6). Only a subgroup of individuals demonstrated, despite high AIs, lower level of Beta detection (<70% of S-Flow; fig. S6). In individuals vaccinated with three doses (V3,1-2M), the ones who displayed greater IgG cross-reactivity against variants were those with the higher AIs (fig. S7). Of interest, vaccinees who received one dose of mRNA-1273 developed IgG with stronger avidity capable to recognize better Omicron than those who received only three doses of BNT162b2 (fig. S7).

For IgA, individuals with two doses were not tested due to the low levels of IgA (Fig. 1D). We found that the IgA AI was high in convalescent individuals boosted with the vaccine (Pre + V: 71 ± 18.9%) compared to convalescents (Pre: 40 ± 15.2%; Fig. 6B). The AIs of vaccinees with three doses are high (79 ± 27.3%) and similar to those observed in convalescents with a boost (Fig. 6B).

Our results from the longitudinal follow-up of the three individuals indicated that IgG avidity declined soon after vaccination, even in individuals who had been previously infected or after two doses (Fig. 6, C to E). Thus, an additional boost was required to improve the avidity. The avidity was similar after three or four doses (Fig. 6E). Thus, these data indicated that the AI decreases rapidly despite repeated vaccinations.

**Fig. 7.**
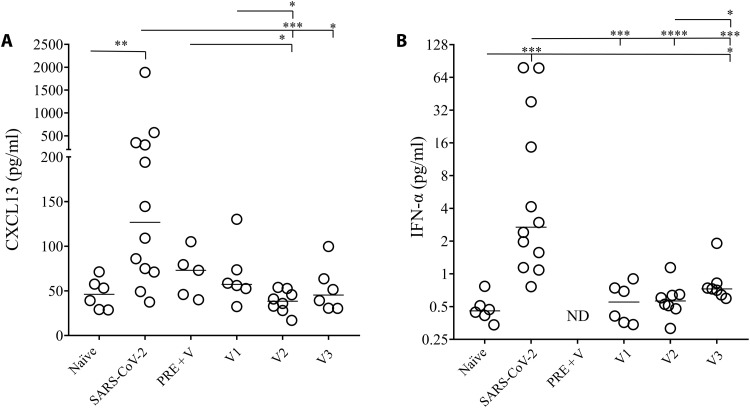
Plasma levels of CXCL13 and type I IFN measurement in healthy donors, acute SARS-CoV-2–infected individuals, and vaccinees. (**A**) CXCL13 and (**B**) interferon-α (IFN-α) levels in the plasma of either healthy donors (naïve), acutely infected individuals (SARS-CoV-2), preexposed vaccinated individuals (Pre + V) and individuals vaccinated with one dose (V1), two doses (V2), or three doses (V3) of vaccine. Blood was collected 15 days after vaccination or infection. Each dot represents one individual. Lines represent median values. Statistical analysis was performed using a Mann-Whitney *U* test (*P < 0.05; **P < 0.01; ***P < 0.001; ****P < 0.0001). ND, not done.

Ig affinity maturation depends on the formation of GC and requires the interaction of B and T cells in B cell follicles (*57*). CXCL13 represents in humans a surrogate marker of GC activation and is associated with neutralizing antibodies with high avidity (*40*–*42*), while humoral response occurring in the extrafollicular zones generates short-lived B cells and Ig with low avidity (*58*, *59*). Furthermore, type I interferon (IFN) may also contribute to the induction of CXCL13 (*60*). Thus, we assessed the levels of CXCL13 in vaccinees from whom samples were obtained within 2 weeks after vaccination including preexposed individuals (Pre + V) and in individuals acutely infected (SARS-CoV-2), compared to healthy individuals (naïve; Fig. 7A). In the analysis of individuals receiving one (V1, 57.3 ± 13.7 pg/ml), two (V2, 38.5 ± 4.4 pg/ml), or three doses (V3, 51.5 ± 10.8 pg/ml), the levels of CXCL13 were similar to those of healthy donors, whereas their levels were higher in acutely infected patients (Fig. 7A). Although not significantly different, the levels of CXCL13 were lower in healthy individuals (46.1 ± 6.9 pg/ml) compared to preexposed individuals boosted by mRNA vaccine (Pre + V, 73.02 ± 11.8 pg/ml). However, because of the limited number of individuals tested, this difference deserves to be further addressed. Similarly, we found that IFN-α levels were low in vaccinees compared to those of acutely infected individuals (Fig. 7B). Therefore, the low levels of CXCL13 are associated with the low avidity observed in vaccinees, suggesting suboptimal GC induction.

Together, these results indicate that boosts are required to improve the avidity of specific IgG, in particular, in individuals whose immunity is only due on vaccination and is associated with low CXCL13 levels. They also suggest that loss of avidity may contribute to the loss of cross-reactivity against the different variants that we observed in vaccinees compared to previously infected individuals and boosted with the vaccine.

## DISCUSSION

Overall, our results indicate that individuals previously infected with SARS-CoV-2 and boosted with mRNA vaccines developed a strong humoral response, whereas multiple doses of vaccines are required to induce similar responses in nonexposed individuals. The immune response induced by the mRNA vaccine has valuable cross-reactivity against the different viral variants, although Beta and the more recent Omicron BA.2 sublineages are of major concern in this perspective, since their lower recognition on the cell surface will also reduce Fc effector functions. Therefore, in the absence of previous infection, repeated administrations of mRNA vaccine are required and can be boosted by a fourth dose, providing a possible explanation for their increased preventive effect on the development of severe disease by different variants (*61*). Unlike IgG, the presence of IgA may also be beneficial in providing protection at the mucosal viral entry (*62*). IgA antibodies were induced only after the third dose. However, unfortunately, because of their short half-life and their low avidity, IgA may confer a protection of limited duration, especially regarding SARS-CoV-2 variants.

Considering the half-life and low avidity of antibodies and the requirement for repeated vaccinations, the low levels of CXCL13 probably indicate suboptimal GC activations and extrafollicular B cells maturation in vaccinated individuals. B cell maturation and Ig avidity leading to high neutralizing antibodies depend on B and T cell interaction, and CXCL13 in the plasma is a surrogate marker of GC activation (*40*–*42*). It has been shown by Samanovic *et al*. (*63*) that the levels of CXCL13 remain low after two doses of vaccine. We confirm this finding and show that, even after three doses of vaccine, they remain similar to those of healthy donors and lower than the levels observed in individuals infected by SARS-CoV-2. This is also consistent with the general absence of hyperplasia of draining lymph nodes following mRNA vaccination (less than 0.3% of the recipients) (*32*). Of interest, other vaccines have been reported to increase the levels of CXCL13 in individuals having received either yellow fever vaccine or Ad5/HIV vaccine (*40*). The absence of type I IFN after BNT162b2 vaccination is consistent with vaccine manufacturing in which RNA has been modified to markedly reduced innate immune sensing and inflammation (*64*) Thus, consistent with the earlier theory of P. Matzinger in 90s as “the danger theory of immunity” (*65*), the absence of innate sensing may require repetitive vaccination for maintaining high levels of antibody. A previous report, using fine needle aspirates of draining axillary lymph nodes, has indicated the presence of GC B cells in vaccinees for 2 months after the boost (*66*); however, this cannot exclude the detection of extrafollicular B cells. Our follow-up study showed that the avidity decreases after 2 months. Kim *et al*. (*67*), performing bone marrow aspirates in individuals vaccinated 6 months earlier with two doses of BNT162b2, have observed that the frequency of bone marrow plasma cells against the S protein was at least 20-fold lower compared to those induced by the 2019-2020 influenza virus vaccine. One limitation of our study is the absence of draining lymph nodes data from vaccinees that may help to elucidate lymphoid organization and GC development, as we previously described for other infectious diseases (*68*, *69*).

In the past, the level and persistence of antibodies were demonstrated as being low, even with repeated immunizations, in the absence of T cell help and associated with extrafollicular B cells (*70*–*72*). Thus, short-lived B cell immunity was counteracted by associating a “carrier,” as a T cell dominant epitope to improve B cell immunity. Moreover, one possibility of improving mRNA vaccines could be to use cytokines such as interleukin-12 that boosts GC formation and could be considered for longer-lasting immune response (*73*). Of interest, in individuals previously infected and boosted with vaccine, the humoral response was extremely rapid and stronger in quantity and quality compared to individuals having received two doses of vaccine. This indicates an imprinting of SARS-CoV-2 immunity in hybrid responders. These results are consistent with the presence of activated memory B cells described by Rodda *et al*. (*74*) in hybrid immunity. This persistence of memory B cells could reflect larger amounts of antigen present after infection and longer ongoing B cell follicle activation contributing to the imprinting, which is consistent with the levels of CXCL13 detected in the plasma of SARS-CoV-2–infected individuals during the acute phase.

We also demonstrated that the avidity of Ig decreased rapidly even after repetitive boosts. This is of importance, because, generally, avidity is associated with the neutralizing capacity of antibodies (*75*). Furthermore, the lower ability of plasma from vaccines to recognize Beta and Omicron variants might also be related to the lower avidity of antibodies induced by vaccination alone. Our results, using the S-Flow assay, are indicative of a lower capacity to recognize the variants, which may also have consequences for humoral response related to cell-mediated cytotoxicity. The ability to eliminate infected cells might contribute to limiting the duration of infection and/or viral dissemination (*76*). In this context, it cannot be excluded that viral spread through cell-to-cell transmission can evade neutralizing antibodies. This is well known in HIV infections (*77*, *78*) and was recently demonstrated in SARS-CoV-2 (*79*). Consistent with a previous report (*25*), we found that convalescent individuals in the absence of a vaccine boost displayed low quality of antibodies capable to recognize the S protein on the surface of cells, as compared to vaccinated individuals. However, our results highlighted that once vaccinated, convalescent individuals develop a strong humoral response with broader IgG cross-reactivities against SARS-CoV-2 variants. Nevertheless, of particular concern is the low recognition of the S protein from Beta and BA.2 Omicron sublineages (BA.2.12.1 and BA.275) by vaccinees’ plasma. Previous reports, on the basis of neutralizing assay using both pseudoviruses and viruses, showed that BA.1 (certainly related to the higher number of mutations in the RBD) was more resistant compared to Beta (*80*, *81*). In contrast, the S-Flow assay indicated that Beta is less recognized than BA.1 Omicron. Our longitudinal analysis also suggests that the quality of antibody is declining after the third dose, over the 6 months of follow-up, particularly regarding the Beta and Omicron variants. Although limited to one individual, we have observed that despite a fourth dose, Beta recognition declined again. Therefore, additional studies deserve to be conducted analyzing the effect of an additional boost or even after viral exposure on the duration of the humoral response. A fourth dose was described to improve protection as compared to three doses of vaccine (*61*). Thus, our results suggest the importance to also assess the capacity of Ig to recognize SARS-CoV-2 variants on cell surface highlighting the role of deletions in the NTD region of new Omicron variants as a virus strategy to escape from the immune response.

There are some additional limitations in our study. Whereas we have observed that if the third vaccination was performed with mRNA-1271, then it induced IgA and improved the quality of IgG recognizing Omicron as compared with BNT162b2 vaccinees only; this was performed on a limited number of individuals. A recent report, however, also suggested a beneficial effect of mRNA-1273 to induce IgA compared to BNT162b2 (*82*). Given the role of mucosal immunity against such viral infection, future studies in larger cohorts are needed.

Despite these limitations, our data provided evidence for potential differences in the quantity and quality of the humoral responses in hybrid immune responders compared to individuals having received three doses of mRNA vaccine and highlighted the interest for analyzing immune response directed against S variant proteins expressed on the cell surface. In conclusion, in the context of the generalized third dose of vaccination, our study provides novel findings regarding the levels of protection and the impact of vaccine strategy to control the dynamics of COVID variants.

## MATERIALS AND METHODS

### Study design and participants

The bioclinical features of patients recruited are given in Table 1. This study was approved by the Ethics Committee of the Île-de-France (EudraCT/IDRCB 2020-A00875-34 and Clinical Trials: NCT04351711, Nîmes University Hospital) and from the Clinical Board and Ethics Committee (ref 69/2020, Hospital de Braga, Portugal). All patients had provided written informed consent. We also analyzed samples obtained longitudinally at different time points after vaccination. Blood was collected, and plasma was obtained after centrifugation was frozen to −80°C.

### IgA and IgG humoral responses

Antibody production was monitored by measuring specific Igs via ELISA against N and S1 proteins as previously described (*16*). Briefly, NUNC MaxiSorp well plates were coated with antigens (0.5 μg/ml in tris-HCl, pH 9.6) overnight. After saturation with bovine serum albumin (BSA), plasmas were diluted to 1:400 and 1:800 and incubated for 90 min. Plates were then washed and incubated with goat anti-human IgG (Fc-specific) peroxidase (A0170, MilliporeSigma) and goat anti-human IgA (Fc-specific) peroxidase (SAB3701229, MilliporeSigma) for 45 min. These antibodies were highly specific to the Fc fragments not recognizing the kappa and lambda chains of the Ig. After several washings, substrate reagent solution (R&D Systems) was added and incubated for 30 min. The reactions were stopped using sulfuric acid (1 N). The plate was read on a Thermo Scientific Varioskan reader at wavelengths of 450 and 540 nm.

### SARS-COV-2 spike avidity assay

Like for ELISA, NUNC MaxiSorp ELISA plates were coated with S1 antigen to monitor the avidity of IgA and IgG. Once incubated in the presence of 1:400 dilution of plasma, plates were washed with phosphate-buffered saline (PBS) and then incubated for 30 min at 37°C in the absence (PBS) or presence of 6M urea. Thereafter, similarly specific IgA and IgG were detected with secondary antibodies and revealed with substrate reagent solution (R&D Systems). The AI was calculated as follows: AI% = (OD value of urea-treated sample/OD of untreated sample)*100. Indexes with values more than 50% were considered as high IgG avidity, 31 to 49% was considered as intermediate IgG avidity, and values below 30% were considered as low IgG avidity.

### S-Flow assay

The assay was conducted in two settings, using SARS-CoV-2–infected or SARS-CoV-2–transfected cells. The day before infection, 4 × 10^6^ Vero-E6 cells were seeded in 75-cm^2^ cell culture flasks in Dulbecco’s minimum essential medium (DMEM) supplemented with 10% heat-inactivated fetal bovine serum (FBS) and penicillin and streptomycin solution (100 μg/ml) and incubated at 37°C and 5% CO_2_. On the day of infection, the cell monolayer was 90% confluent. Medium was removed, cells were washed once with medium, and different flasks were inoculated with: SARS-CoV-2 Wuhan-Hu strain (Global Initiative on Sharing All Influenza Data accession no. EPI_ISL_16833248) at a multiplicity of infection of 0.01. Cells were incubated for 1 hour at 37°C with shaking. The inoculum was then replaced with DMEM containing 2% FBS. Two days after inoculation, cells were harvested with trypsin (Gibco) and centrifuged for 3 min at 900*g*. In the other setting, 293T cells were transfected using Lipofectamine 2000 (Life Technologies) and plasmids encoding the full length of the SARS-CoV-2 S variants (*47*). The Wuhan-Hu S-expressing plasmid was provided by O. Schwartz, whereas Beta and Delta were purchased from InvivoGen (Spike pseudotyping plasmid, plv-spike-v3 and plv-spike-v8, respectively), and the Omicron S protein (BA.1 and BA.2 sublineages) plasmids were produced in-house. After transfection and overnight culture, the cells were detached using PBS-EDTA and transferred into U-bottom 96-well culture plates (200,000 cells per well). For both infected and transfected cells, the cellular pellets were saturated with 10% FBS at 4°C for 10 min and incubated with the patients’ plasma (1:300 dilution) in PBS containing 0.5% BSA for 30 min at 4°C. Cells were then washed and stained for 30 min at 4°C using the same antibodies as described for ELISA but labeled with fluorescein isothiocyanate (Sigma-Aldrich). After washing, cells were fixed with 2% paraformaldehyde. Furthermore, we used two mAbs as controls in this study: anti-spike (S2) (GeneTex, clone 1A9) and anti-NTD mAb (ProteoGenix, clone 4A8). The mAbs were diluted to 1:1000 and revealed using specific Alexa Fluor 488–labeled secondary antibodies. Cells were analyzed on an Attune NxT flow cytometer using FlowJo software (Tree Star Inc.).

### Quantification of CXCL13 and IFN-α

The amounts of CXCL13 and IFN-α in the plasma were quantified by ELISA (R&D Systems). Plates were read at a reference wavelength of 490 nM.

### Statistical analyses

Statistics were calculated using GraphPad Prism software. A nonparametric Mann-Whitney *U* test and Student’s *t* test were used for comparison. *P* values indicate significant differences (*P < 0.05; **P < 0.01; ***P < 0.001; ****P < 0.0001). Correlations were assessed using the Spearman test. A chi-square test was used to compare frequency.
