# Supplementary material for: Repetitive mRNA vaccination is required to improve the quality of broad-spectrum anti–SARS-CoV-2 antibodies in the absence of CXCL13
Source: Sci Adv. 2023 Aug 4;9(31):eadg2122. doi: 10.1126/sciadv.adg2122 (PMC10403221; doi:10.1126/sciadv.adg2122)
Supplement: Supplementary file 1 — Figs. S1 to S7 [file sciadv.adg2122_sm.pdf]

Science Advances
AAAS

Supplementary Materials for

Repetitive mRNA vaccination is required to improve the quality of broad-spectrum anti-SARS-CoV-2 antibodies in the absence of CXCL13

Marne Azarias Da Silva *et al.*

Corresponding author: Jérôme Estaquier, jerome.estaquier@crchudequebec.ulaval.ca

Sci. Adv. **9**, eadg2122 (2023)
DOI: 10.1126/sciadv.adg2122

This PDF file includes:

Figs. S1 to S7

Supplementary Text

Figure S1. IgG response against the spike protein in convalescents and vaccinated individuals. Plasma from convalescent individuals (Pre), convalescent individuals boosted with vaccine (Pre+V), vaccinees after two doses either at months 1 to 3 (V2,1-3M) or months 4 to 6 (V2,4-6M) and after three doses at months 1 to 2 (V3,1-2M) are diluted to 1/800. Specific IgG were tested against spike (S) protein. OD, optical density, are shown. Each circle represents one individual. Lines represent median values. Dashed lines represent antibody specificity (0.25 OD) in comparison to IgG from healthy donors. Statistical analysis was performed using a Mann-Whitney *U* test (**, $p<0.01$; ****, $p<0.0001$).

Figure S2. Binding of anti-SARS-CoV-2 antibodies to infected cells.
(A) Vero-E6 cells either not infected or infected with SARS-CoV-2, were incubated with plasma from a healthy donor (HD), or a convalescent individual boosted with a vaccine dose (Pre+V), or a monoclonal antibody against the S protein (mAb). The percentages of cells expressing viral proteins on their surface, and recognized by plasma IgG or by the mAb were determined by flow cytometry using the S-Flow assay. (B) Vero-E6 cells either non infected (left) or infected by SARS-CoV-2 (right) were incubated with plasma from a representative panel of vaccinees and convalescent individuals, and analyzed by the S-Flow assay. The percentage of positive cells recognized by specific IgG is shown. Statistical analysis was performed using a Mann-Whitney *U* test (****, $p<0.0001$).

Figure S3. Monoclonal anti-spike (S2) against SARS-CoV-2 viral variants.
HEK293T cells transfected with a plasmid encoding for the S protein for different SARS-CoV-2 Strains were detected using the anti-IgG mAb directed against the S2 spike region. S-Flow assays of (A) Wuhan-Hu, (B) Delta, (C) Beta and (D) Omicron are shown.

Figure S4. Structural variations in the RBD and NTD regions of Beta, Delta, and Omicron (BA.1 and BA.2) variants.
(A) Root mean square (rms) deviation after structural alignment with the Wuhan-Hu RBD as a reference for Beta, Delta, Omicron BA.1 and BA.2. All RBD are in the up conformation and in interaction with an ACE2 molecule (PDB_ID used: 7KJ4 (Wuhan), 7VXM (Beta), 7V8A (Delta), 7T9K (Omicron BA.1) and 7XOA (Omicron BA.2)). (B) The RBD superimposed structures are shown in the interaction with ACE2 (from PDB_ID 7KJ4). Rms deviation after structural alignment

with the Wuhan-Hu NTD as a reference against Delta (C), Beta (D), Omicron BA.1 (E) and BA.2 (F). Black bars represent inserted/deleted regions.

Figure S5. Monoclonal anti-NTD against SARS-CoV-2 viral variants.
Representative S-Flow assay of HEK293T cells transfected with a plasmid encoding for the S protein for different SARS-CoV-2 Strains (Wuhan-Hu, Delta, Beta and Omicron BA.1) and detected by flow cytometry using anti-NTD IgG mAb.

Figure S6. Correlation between IgG cross-reactivity and avidity indexes in vaccinated pre-exposed individuals.
Figures show the avidity indexes of IgG from pre-exposed and vaccinated individuals against the percentages of variant recognition: (A) Wuhan-HU, (B) Delta, (C) Beta, and (D) Omicron B.A.1 strains. The values are from figures 2 and 6. Symbols with a cross represent individuals who received at least one dose of mRNA-1273 whereas open symbols represent individuals who only received BNT162b2 in the vaccination scheme.

Figure S7. Correlation between IgG cross-reactivity and avidity indexes in vaccinated naive individuals.
Figures show the avidity indexes of IgG from vaccinated individuals against the percentages of variant recognition: (A) Wuhan-HU, (B) Delta, (C) Beta, and (D) Omicron B.A.1 strains. The values are from figures 2 and 6. Symbols with a cross represent individuals who received at least one dose of mRNA-1273 whereas open symbols represent individuals who only received BNT162b2 in the vaccination scheme.

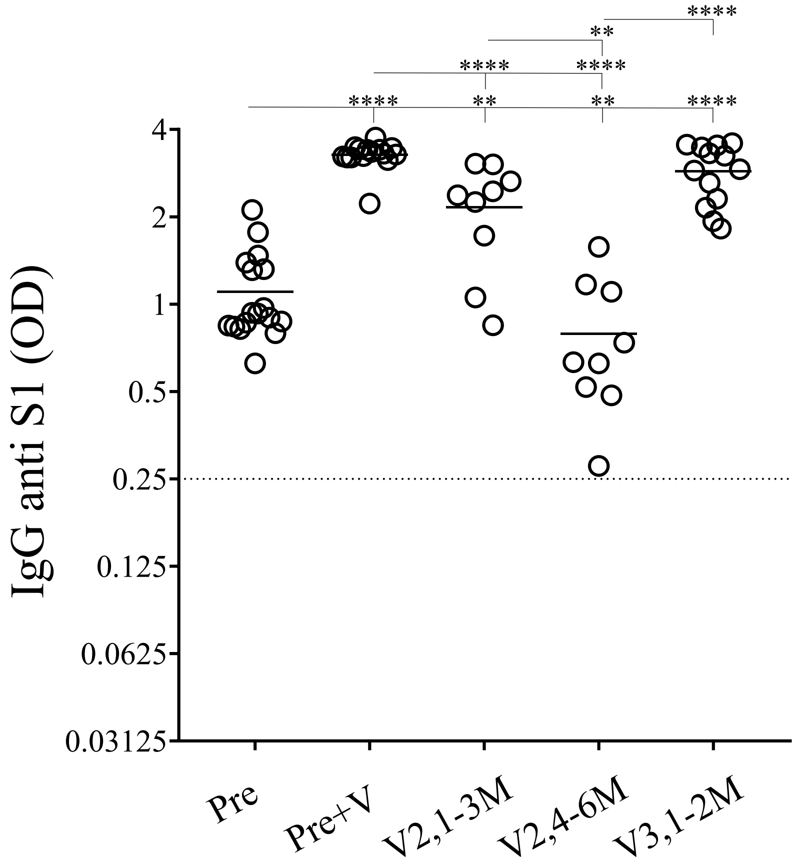

Anti-S (1/800)

Figure S1

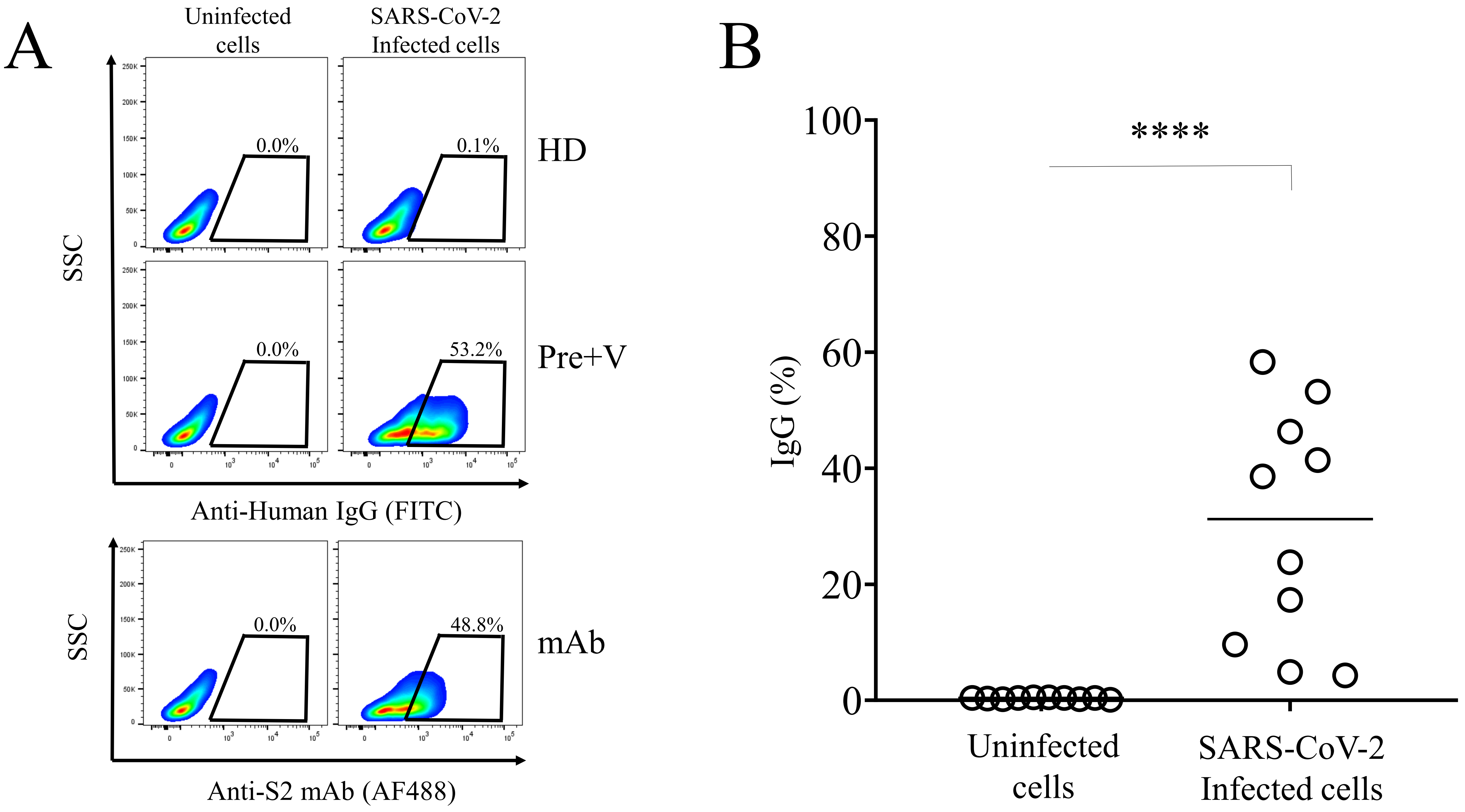
A
Uninfected cells
SARS-CoV-2 Infected cells
HD
Pre+V
mAb
SSC
0.0%
0.1%
0.0%
53.2%
0.0%
48.8%
Anti-Human IgG (FITC)
Anti-S2 mAb (AF488)
B
IgG (%)
****
100
80
60
40
20
0
Uninfected cells
SARS-CoV-2 Infected cells

Figure S2

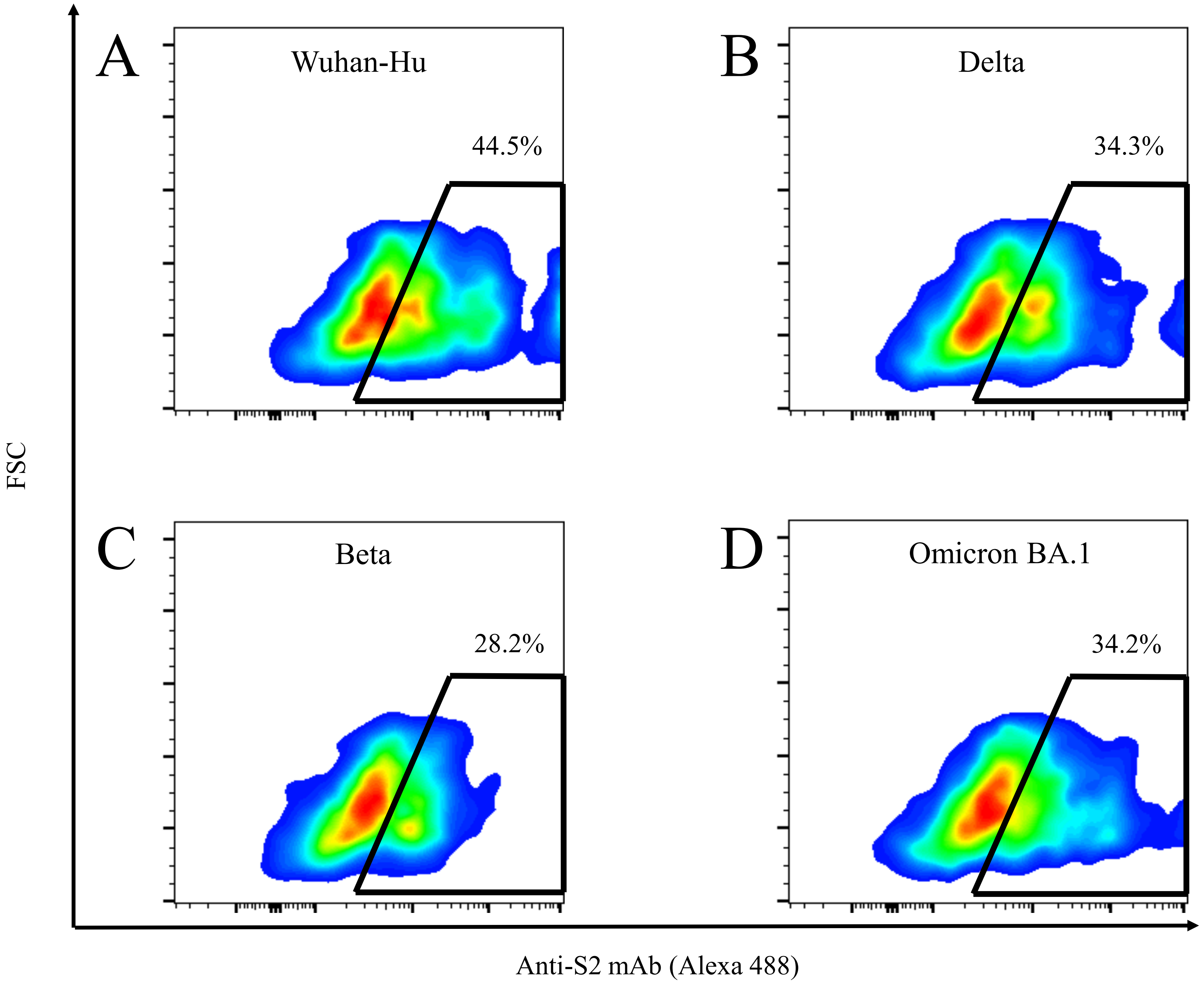
A
Wuhan-Hu
44.5%
B
Delta
34.3%
C
Beta
28.2%
D
Omicron BA.1
34.2%
FSC
Anti-S2 mAb (Alexa 488)

Figure S3

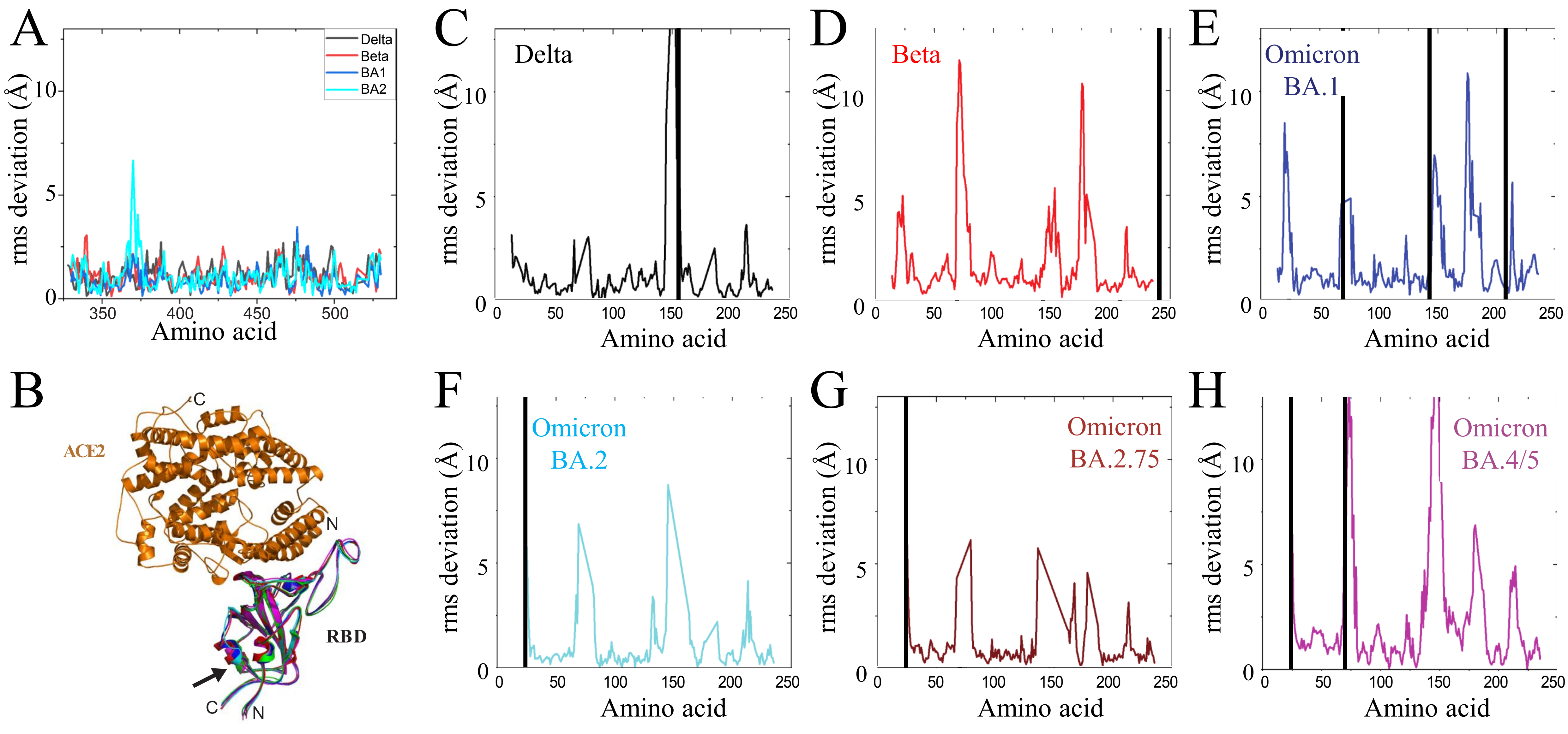
A
rms deviation (Å)
Amino acid
Delta
Beta
BA1
BA2
B
ACE2
RBD
C
N
C
Delta
D
Beta
E
Omicron
BA.1
F
Omicron
BA.2
G
Omicron
BA.2.75
H
Omicron
BA.4/5

Figure S4

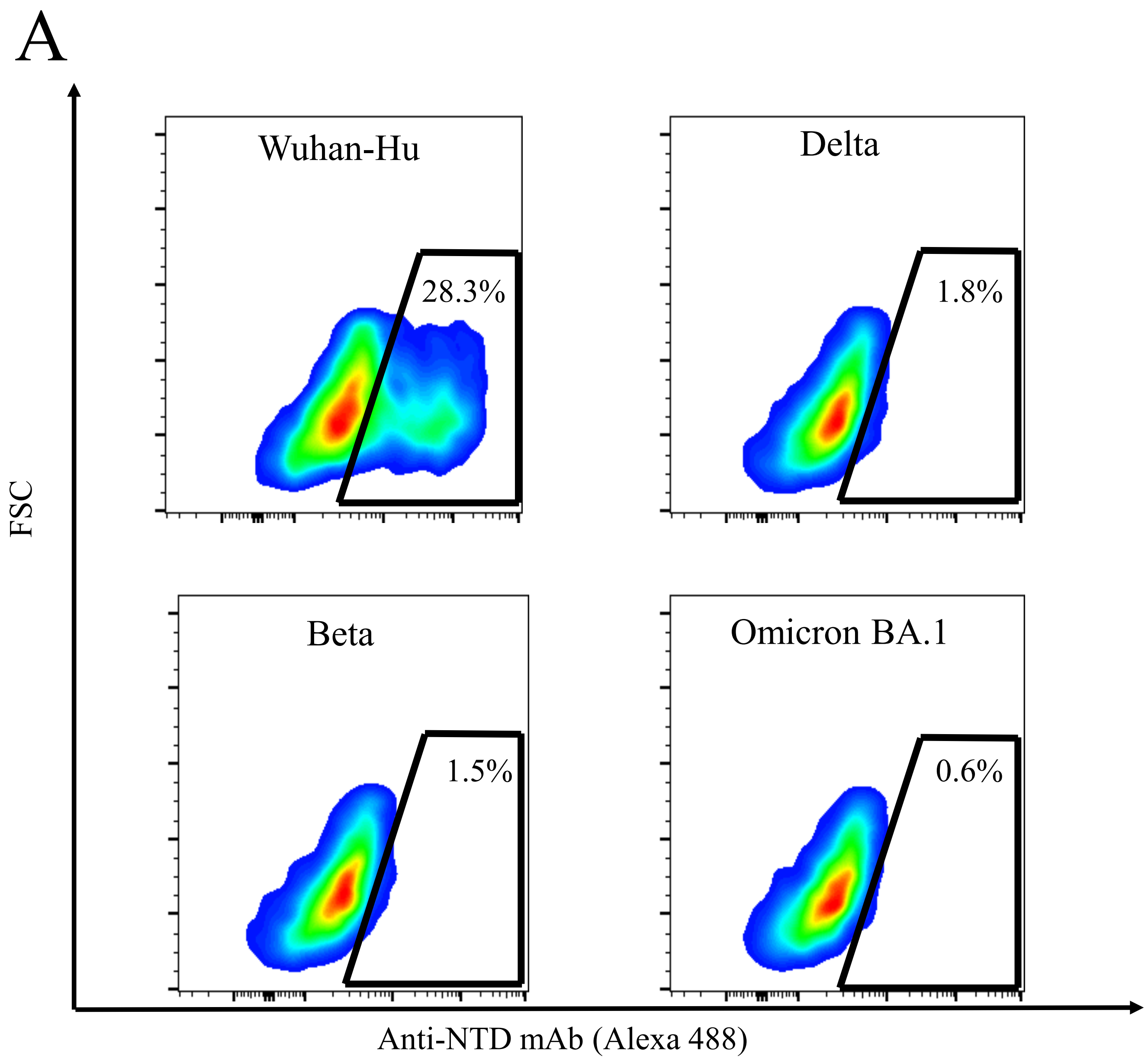
A
Wuhan-Hu
28.3%
Delta
1.8%
Beta
1.5%
Omicron BA.1
0.6%
FSC
Anti-NTD mAb (Alexa 488)

Figure S5

Pre+V

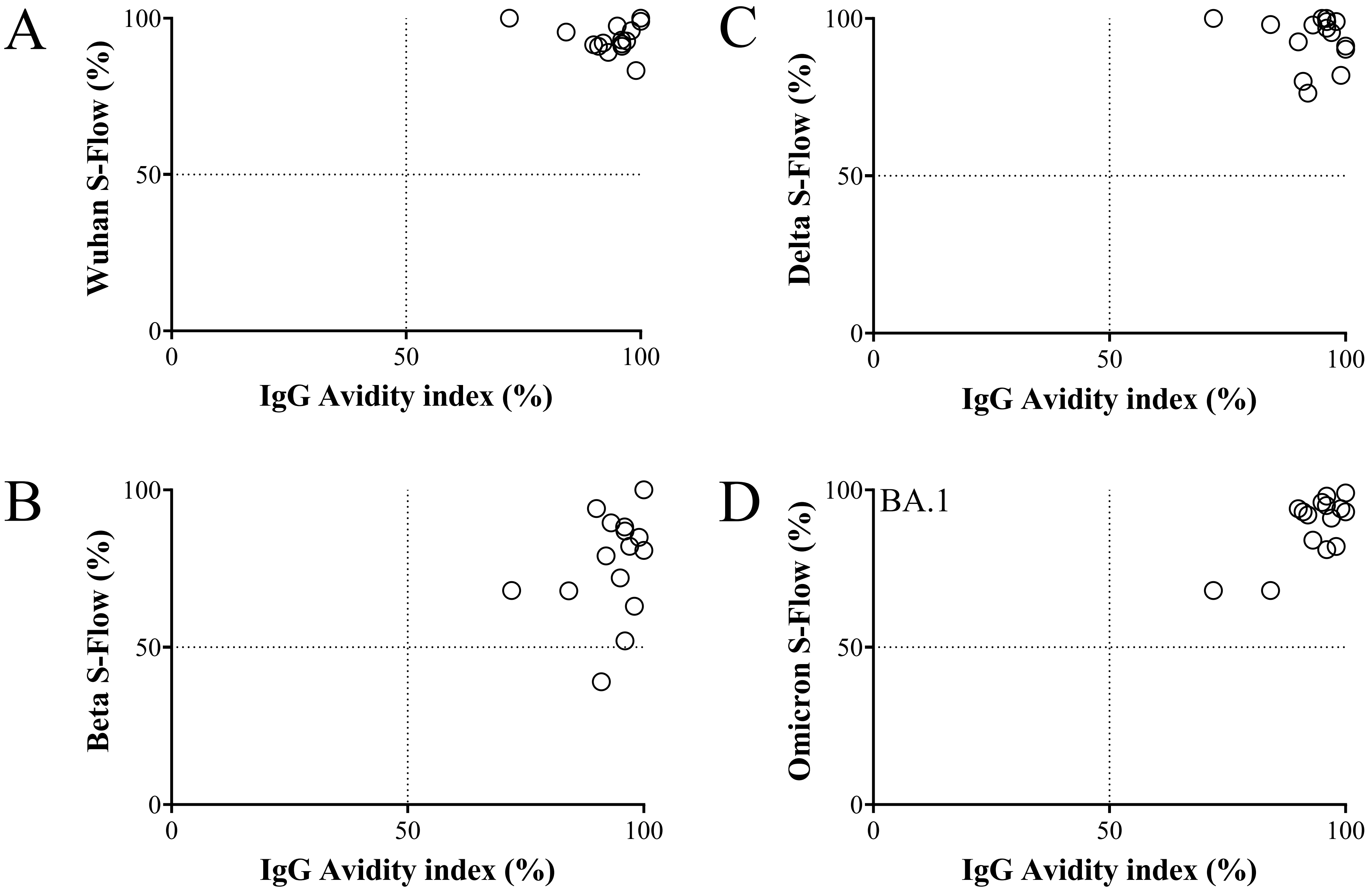

Figure S6

V3 – 1-2M

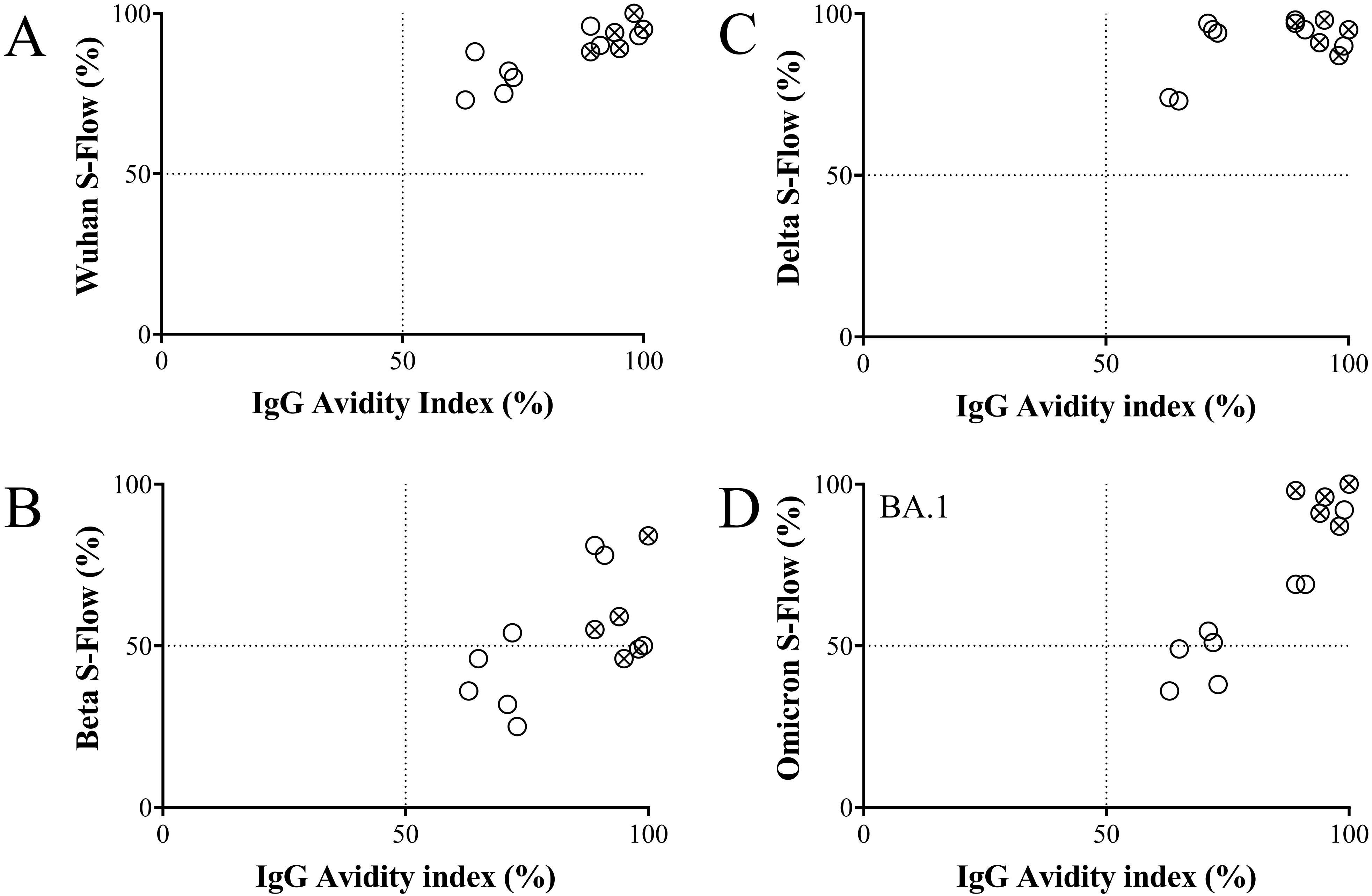

Figure S7
